# Verbal Weight‐Related Abuse and Binge Eating Behavior: The Mediating Role of Attentional Bias to Threat Cues and Difficulties in Emotion Regulation

**DOI:** 10.1002/brb3.70054

**Published:** 2024-09-30

**Authors:** Elnaz Salemi, Shaghayegh Zahraei, Gholamreza Dehshiri, Abdolreza Norouzy

**Affiliations:** ^1^ Department of Psychology Faculty of Education and Psychology Alzahra University Tehran Iran; ^2^ Department of Nutrition Mashhad University of Medical Sciences Mashhad Iran

**Keywords:** attentional bias to threat cues, binge eating behavior, difficulties in emotion regulation, verbal weight‐related abuse

## Abstract

**Introduction:**

Previous studies investigated the impact of weight‐related abuse (WRA) on eating pathology. However, the circumstances of such an effect are still unclear. Our study aimed to examine the relationship between verbal WRA and binge eating (BE) behavior via attentional bias (AB) to threat cues and difficulties in emotion regulation.

**Method:**

We conducted a parallel mediation model. On the basis of the purposive sampling method, 183 individuals with obesity and overweight (70.5% female and 28.4% male; Mean_age_ = 32.78), from February to June 2019, were recruited from a nutrition clinic in Tehran. The participants completed the BE scale (BES), the weight‐related abuse questionnaire (WRAQ), the difficulties in emotion regulation scale (DERS), and the dot probe task (DPT).

**Results:**

AB to threat cues had a significantly negative association with verbal WRA and BE. Difficulties in emotion regulation showed a significant positive association with verbal WRA and BE. The parallel mediation model showed a direct effect of verbal WRA on BE. Moreover, the bootstrap analysis revealed that difficulties in emotion regulation could mediate the association between verbal WRA and BE.

**Conclusions:**

Our findings suggest that experiences of verbal WRA can contribute to cognitive bias to negative emotion, maladaptive emotion regulation strategies, and behavioral problems like BE.

## Graphical abstract

This study examined the relationship between verbal weight‐related abuse (WRA) and binge eating behavior (BE) through attentional bias (AB) to threat cues and difficulties in emotion regulation. The parallel mediation model revealed that verbal WRA had a direct effect on BE. Although difficulties in emotion regulation could mediate the association between verbal WRA and BE, AB to threat cues could not mediate this relationship.

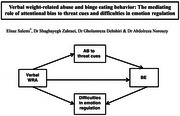

## 1 Introduction

Binge eating (BE) behavior is defined as eating a large amount of food in a short period without having control over it (American Psychiatric Association 2013). Various factors, such as negative affect, thin‐ideal internalization, and perfectionism (Stice 2002), are associated with a high risk of BE. Early experiences of sexual, physical, and emotional/verbal abuse can also play a crucial role in the development and maintenance of BE (Burns et al. 2012; Imperatori et al. 2016; Caslini et al. 2016). However, the effect of emotional/verbal abuse on eating disorders has gained considerable attention (Burns et al. 2012; Mills et al. 2015), and it is indicated to be a significant contributor to eating pathologies (Salwen et al. 2015).

One of the emotional/verbal maltreatment is weight‐related teasing. A longitudinal study indicated that 42% of females and 44% of males experienced weight‐related teasing in their early adolescence (Haines et al. 2008). Although weight‐related teasing has been the focus of many previous studies, the concept of weight‐related abuse (WRA; i.e., “significant verbal or physical victimization or maltreatment specific to one's weight”; Salwen and Hymowitz 2015) has been developed recently. We, in this study, aimed to evaluate verbal WRA because it measures a diverse range of verbal weight victimization, from teasing to more serious maltreatment, including “being threatened” because of one's weight (Salwen and Hymowitz 2015). Studies show verbal WRA predicts the development of subsequent BE (Salwen et al. 2015). However, not all people facing verbal WRA report future BE (Bannon, Salwen, and Hymowtiz 2018), and the underlying factors that might theoretically interfere in the relationship between verbal WRA and BE need further exploration.

Hunger et al. (2015) suggest that disturbance in self‐regulatory resources and individuals’ efforts to avoid weight stigma are related to problematic health behaviors. In line with this finding, the Stigma Control Model of Dysregulated Eating (Mason, Smith, and Lavender 2018) offers a pathway from victimization toward dysregulated eating behaviors through stigma management strategies. In other words, this model posits that experiencing stigma about features, such as race, sexuality, weight, and appearance, might lead to cognitive and emotional stigma management processes (Mason, Smith, and Lavender 2018; Hatzenbuehler 2009), such as avoidance, rumination, and vigilance about negative emotions (Mason, Smith, and Lavender 2018). Therefore, we suppose that people with the experiences of weight discrimination may become more sensitive to negative and threatening emotions. In other words, maltreatment experiences can affect threat information processing (Gibb, Schofield, and Coles 2009). The reason for this influence is related to the limited capacity of perceptual and sensory systems, which possibly leads individuals to filter the processed information based on their experiences. When the environment is abusive, threatening messages can be perceived as salient cues (Pollak and Tolley‐Schell 2003), or they might be frequently avoided as a temporary function to decrease distress (Humphreys et al. 2016). Hence, one specific self‐regulation mechanism that people with experiences of verbal WRA may deploy is to divert their attention toward or away from negative emotions, defined as attentional bias (AB) to threat cues.

AB to threat cues means the level of individual engagement, disengagement, or avoidance from threatening stimuli (Stojek et al. 2018). The processing of threatening stimuli shows that once the strength of threat is detected, people either devote their attention toward the threat and respond to it with engagement or avoid the situation (Beck and Clark 1997). Some researchers maintain that people dealing with eating problems might engage with the threat (MacManus, Waller, and Chadwick 1996; Morrison 2005; Starzomska 2017), and some others argue that they more probably employ attentional avoidance to escape from threatening stimuli (Deroos and Cserjési 2018; Davies et al. 2011). However, as BE is considered to be one of the mechanisms to avoid negative emotional states (Heatherton and Baumeister 1991), an association between BE and attentional avoidance is expected, in this study. On the basis of the literature mentioned above, AB to threat cues could be assessed as a possible mediator in the relationship between verbal WRA and BE.

In addition to AB to threat cues, difficulties in emotion regulation could be considered another potential mediator in the relationship between verbal WRA and BE. One's ability to effectively manage emotions develops through a secure early environment. Therefore, an emotionally abusive environment can adversely affect the regulation of emotions (Mills et al. 2015). Moreover, previous findings show that maltreatment experiences are significantly associated with later poor ability to regulate emotions (Gruhn and Compas 2020). Additionally, the relationship between difficulties in emotion regulation and dysregulated eating has been established before. In other words, individuals with BE appear more prone to emotion regulation difficulties (Svaldi et al. 2012; Harrison et al. 2010). Moreover, it has been suggested that individuals might engage in BE as a regulatory strategy to escape from compound emotional distress (Heatherton and Baumeister 1991). Previous studies have also demonstrated that the dysregulation of negative emotions arising from emotional abuse, discrimination, and stigma can sometimes encourage more complex negative emotional states and further behavioral difficulties such as maladaptive eating behavior (Mason, Smith, and Lavender 2018; Hatzenbuehler 2009; Mills et al. 2015).

Overall, previous studies have established the links between different sorts of abuses and AB to threat cues, difficulties in emotion regulation, and BE. However, these relationships in terms of verbal WRA and their interplay toward BE are underexplored. Additionally, studying verbal WRA in Iran might be important because there are limited studies on weight‐related discrimination. Moreover, weight‐related maltreatment appears to be a more significant risk for people living in developing countries (Hackman, Maupin, and Brewis 2016) such as Iran. Furthermore, regarding eating problems, many young Iranians are reportedly at risk of eating disorders (Rauof et al. 2015). Therefore, considering all the abovementioned cases, we supposed that studying the contributing factors in the relationship between these two high‐risk issues (weight‐related maltreatment and eating problems) could hopefully serve as a basis for further research in our population.

### 1.1 Current Study

On the basis of the literature provided above, the purpose of this study was to investigate the association between verbal WRA and BE. Furthermore, we examined the possible mediating role of AB to threat cues and difficulties in emotion regulation in the relationship between verbal WRA and BE.

## 2 Methods

### 2.1 Participants

The population of this study included individuals with overweight and obesity (body mass index [BMI] of 25–29.9 and BMI of 30 or higher, respectively) defined by the National Institute of Health (1998). We performed the statistical *F* test (linear multiple regression: fixed model, *R* ^2^ deviation from zero) in the G*Power software program v.3.1 (Faul et al. 2009) to estimate the minimum sample size. The program suggested at least 119 participants to reach a power of 0.95 and a medium effect size of 0.15 (Cohen 1988). Therefore, from February to June 2019, 185 individuals with obesity and overweight (National Institute of Health 1998), on their first appointment for diet‐based therapy, were recruited from the Nutrition Ward of a Gastroenterology and Hepatology clinic located in Tehran. BMI (kg/m^2^) of 25 or higher calculated by the electronic weight/height scale, and age over 21 were the inclusion criteria. Furthermore, self‐report current/history of mental (e.g., intellectual disabilities) and physical disorders (e.g., diabetes) affecting the participant's capacity to comply with the research and influencing their weight were the exclusion criteria. Because none of the participants reported any serious conditions, there were no exclusions as a result.

All study participants provided written informed consent. Moreover, the ethical standards were approved by the Research Committee of a public university in Tehran (IR.MODARES.REC.1397.086).

### 2.2 Measures

Weight‐related abuse questionnaire (WRAQ; Salwen and Hymowitz 2015) is a 15‐item measure designed to assess the experience of weight‐related verbal (8 items) and physical (7 items) maltreatment and their perceived emotional impacts before the age of 21. Items are rated on a 6‐point Likert scale (0 = never, 6 = >20 times per year). Only the verbal subscale was used in this study (e.g., verbal WRAQ‐item1: someone laughed at you because of your weight; verbal WRAQ‐item2: someone called you names because of your weight). We translated this questionnaire into Persian. Current Cronbach's alpha for this subscale was 0.84.

BE scale (BES; Gormally et al. 1982) is a 16‐item scale designed to assess the severity of BE. Items are scored in a range of 0–3. The higher overall score represents the increased severity of BE. People who score 18–26 are considered to have moderate BE, and people who score 27 or above are considered to have severe BE. The Persian translation of this scale shows a high internal consistency (Dezhkam et al. 2009). The present study revealed that Cronbach's alpha was 0.86 for this scale.

Dot probe task (DPT; MacLeod, Mathews, and Tata 1986) is an instrument designed to examine the AB based on the reaction time to different kinds of cues. We used threatening (fearful/angry) and neutral facial expression pictures to assess AB toward/away from the threat. In this task, 42 pairs (threatening and neutral) of pictures emerge on the two sides of the computer screen, each pair for 500 ms. Followed by these pictures, the participants indicate the place of a star appearing on one side of the screen via the left/right key on the keyboard. In 21 randomly chosen trials, the star would replace the threatening faces (congruent trials), and in the other 21 trials, the star would replace the neutral faces (incongruent trials). AB score was computed by subtracting the mean reaction time of congruent trials from that of the incongruent trials. A positive AB score indicates AB toward threat, and a negative score denotes the AB away from threat. Dehghani, Khatibi, and Pour Etemad (2009) examined the validity and reliability of this task in the Iranian population. In the present study, the split‐half reliability of DPT was measured using the first‐half/second‐half split method (adjusted *r* = 0.12).

Difficulties in emotion regulation scale (DERS; Gratz and Roemer 2004) is a 36‐item scale used to assess difficulties in emotion regulation. Items are rated on a 5‐point Likert scale (1 = almost never, 5 = almost always). The higher total score represents higher difficulties in emotion regulation. Current Cronbach's alpha for this scale is 0.91. Moreover, the Persian translation of this scale shows a high internal consistency (Besharat 2018).

### 2.3 Data Analysis

**FIGURE 1:**
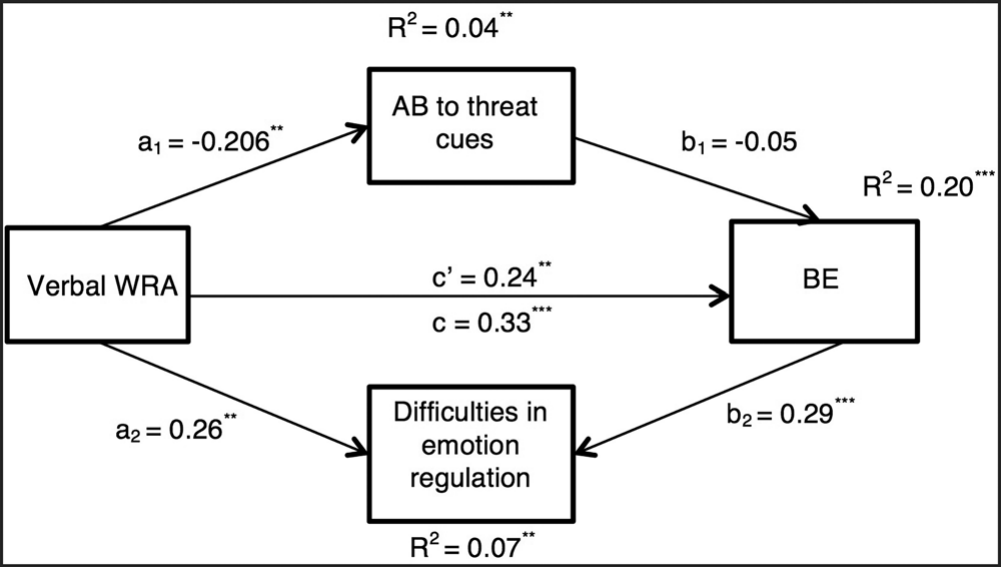
Parallel mediation model (standardized regression coefficients with *p < 0.05, **p < 0.01, and ***p < 0.001). AB, attentional bias; BE, binge eating; WRA, weight‐related abuse.

The SPSS software v.24 was used to analyze the results. The relationship among the variables was investigated through the Pearson correlation. Next, the parallel mediation analysis (Hayes 2017) was performed to study the indirect effect of verbal WRA on BE via AB to threat cues (*a*_1_–*b*_1_ path; Figure 1) and difficulties in emotion regulation (*a*_2_–*b*_2_ path; Figure 1). Point estimates for direct and indirect effects and bias‐corrected confidence intervals (95% BC CIs and 5000 bootstrap replacements) were examined through the bootstrap method. Non‐inclusion of zero for BC CIs would be defined as a significant indirect pathway.

Because BMI, gender, and age were entered into the model as the study covariates but did not change the results, the analysis was repeated without considering the covariates.

## 3 Results

### 3.1 Preliminary Analysis

The missing data analysis indicated no missing data, as participants completed the questionnaires thoroughly. When questionnaires were not completed, evaluators required participants to fill out any missing sections. For the DPT score analysis, the entire datasets of participants with reaction times <100 and >1500 ms for any of the 42 trials were excluded. This exclusion criterion resulted in the removal of 1% of the sample. Therefore, the data of 183 participants (70.5% female, 28.4% male; 62% married, 38% unmarried) with an age range of 21–48 years (*M* = 32.78, SD = 6.91) were analyzed. The average BMI with a range of 25–64.25 was 31.91 (37.2% overweight, 62.8% obesity). Moreover, 64% of the participants reported experiences of verbal WRA, and 38% showed signs of moderate to severe BE.

Table 1 presents the mean, standard deviation, and associations among the study variables. The bivariate correlations between verbal WRA and BE, verbal WRA and difficulties in emotion regulation, and BE and difficulties in emotion regulation were significantly positive. Furthermore, AB was significantly and negatively associated with BE, verbal WRA, and difficulties in emotion regulation.

**TABLE 1:** Means and standard deviations and correlations among the study variables.

	*M*	SD	Range	1.	2.	3.	4.	5.
1. BMI	31.91	5.41	25–64.25	—	0.25^**^	0.16^*^	−0.10	0.03
2. Verbal WRA	7.75	9.04	0–36	—	—	0.33^**^	−0.21^**^	0.26^**^
3. BE	14.98	9.03	0–41		—	—	−0.17^**^	0.37^**^
4. AB to threat cues	−0.0022	0.04	−0.11 to 0.16				—	−0.23^**^
5. Difficulties in emotion regulation	87.24	20.84	40–151					—

Abbreviations: AB, attentional bias; BE, binge eating; BMI, body mass index; WRA, weight‐related abuse.

^*^ *p *< 0.05.

^**^ *p *< 0.01.

As mentioned in the method section, our WRAQ examines verbal WRA in addition to its perceived emotional impact. However, we could not enter the emotional impact score as a separate variable owing to its high positive correlation with verbal WRA (*r *= 0.90).

### 3.2 Parallel Mediation Model

Verbal WRA, AB to threat cues, and difficulties in emotion regulation could predict 20% of the variance in BE (*R* ^2^ = 0.20, *F*(4,178) = 14.64, *p *< 0.001). (See Figure 1 for standard path coefficients).

Findings suggested that the total effect of verbal WRA on BE was statistically significant (*b *= 0.33, *SE *= 0.07, *t*(181) = 4.68, *p *< 0.001). After adding the mediators to the model, the direct effect of verbal WRA on BE was still significant (*b *= 0.24, *SE *= 0.07, *t*(179) = 3.43, *p *< 0.05). The indirect effect of verbal WRA on BE through AB to threat cues was not significant (*b *= 0.01, *SE *= 0.01, 95% BC CI [−0.01 to 0.04]). However, there was a significant indirect effect from verbal WRA on BE via difficulties in emotion regulation (*b *= 0.07, *SE *= 0.03, 95% BC CI [0.02–0.14]). The total indirect effect of this pathway was also significant (*b *= 0.09, *SE *= 0.03, 95% BC CI [0.03–0.16]).

## 4 Discussion

The present study aimed to examine the relationship between verbal WRA and BE through AB to threat cues and difficulties in emotion regulation. As expected, verbal WRA was positively associated with BE, suggesting that individuals exposed to verbal WRA may be at higher risk for BE. This finding provides more support for previous research (Salwen et al. 2015).

The addition of AB to threat cues in our model was negatively related to verbal WRA and BE. Meaning that the higher scores in verbal WRA and BE are associated with donating attention away from threat cues. In other words, verbal WRA and BE are more related to avoidance mechanisms rather than engagement in our sample. In line with previous studies (Mason, Smith, and Lavender 2018), when individuals face maltreatment, they may allocate their attention away from a specific situation (Gross 1998). This process can be helpful to maintain emotional stability during a threatening situation (Johnson 2009). Moreover, we maintain that BE is more associated with attentional avoidance as BE might be considered an escape attempt to draw less attention to negative emotions (Heatherton and Baumeister 1991). To the best of the authors’ knowledge, most of the previous studies have examined AB to threat cues as a comparison criterion between groups of people with eating disorders (Stojek et al. 2018) and reached inconsistent results regarding the kind of AB in these individuals. However, the type of AB is related to the duration of threat processing. In the beginning, recognition/engagement with threats occurs. Nevertheless, after initial recognition, the avoidant mechanisms can be activated (Meyer, Waller, and Watson 2000). Correlation studies examining the association between AB and one specific behavior, such as BE, may shed new light on previous inconsistent results.

Moreover, our findings revealed that AB to threat cues could not mediate the relationship between verbal WRA and BE. Specifically, although there is a significant link from verbal WRA to AB, the pathway from AB to BE is not significant (Figure 1). Regarding our results, we assume that the allocation of attention away from threatening emotions in response to maltreatment is not connected to BE because the mean and variance of AB in our sample were not high compared to previous studies reporting the mean of AB in their samples. This suggests that the scores from congruent trials and incongruent trials were close; thus, most of the sample did not reveal considerable AB to threat cues. In other words, we believe attentional avoidance would be adaptive as long as it is not rigidly used and one's ability to regulate emotions is maturely developed (Bardeen and Daniel 2017). Nevertheless, prolonged attentional avoidance may be able to predict more intense negative emotional states and an increase in behavioral problems such as BE (Milojevich, Norwalk, and Sheridan 2019). Therefore, this path may only be significant in the presence of difficulties in attentional processes.

Expectedly, difficulties in emotion regulation could mediate the relationship between verbal WRA and BE. Consistent with our result, previous studies show that an abusive environment and early exposure to psychological distress (Pollak 2008; Corstorphine 2006) can predict the dysregulation of complicated negative emotions and possibly lead to BE as a response to block intensive distress (Corstorphine 2006). Moreover, on the basis of the model proposed by Mason, Smith, and Lavender (2018), the dysregulated negative emotions arising from verbal victimization and maladaptive strategies to regulate these emotions can increase the risk of BE (Svaldi et al. 2012). The finding is also in line with the works of Mills et al. (2015), Michopoulos et al. (2015), and Burns et al. (2012), testing the mediation role of emotion dysregulation in the relationship between emotional/verbal abuse and pathological eating behaviors. However, as mentioned earlier, there is a lack of exploration of verbal WRA as a unique form of verbal abuse (which cannot be replaced by the general form of verbal abuse) and its impact on BE and difficulties in emotion regulation. Future research should further investigate the development of BE in people with experiences of verbal WRA and their pathways with other potential mediators.

### 4.1 Limitations

We collected our samples from only one nutrition clinic, which affects the generalizability of our results to the community with overweight/obesity. Moreover, the number of male participants was limited, preventing comparison between the two sexes. For example, the previous studies on sex differences regarding BE (Striegel‐Moore et al. 2009; Saccaro et al. 2023) and emotion dysregulation (Ritschel et al. 2015; Lafrance Robinson et al. 2014) showed mixed results. Therefore, future research is suggested to consider sex in examining the variables and pathways presented in the current research.

Our data were collected based on self‐reported measurements, which can increase the risk of biased data. In addition, this study aimed to examine the impact of past experiences of verbal WRA on current cognitions, emotions, and behaviors. Therefore, an important limitation of our research is the cross‐sectional nature of our data, which means that temporal and causal inferences cannot be made about the relationships among the variables. Hence, a longitudinal study would be more consistent with our purposes.

Regarding the use of DPT to measure AB, some research studies have reported poor reliability for this task (Cabrera, Brugos, and Montorio 2020; Ren et al. 2020; Schmukle 2005), a result that was also observed in the current study. However, on the basis of the research by MacLeod, Grafton, and Notebaert (2019), although DPT seems to show low consistency in detecting individual differences in AB, it can still be useful for gathering data from groups. Therefore, despite its limitation for measuring individual differences, DPT can still help researchers examine the relationships between AB and other variables within a group. Nevertheless, future studies are suggested to employ DPT in multiple sessions or to use several measures of AB (e.g., eye tracking) to enhance the reliability of their results (MacLeod et al. 2019; Cabrera et al. 2020).

In this study, we focused on BE as opposed to BE disorder. Therefore, the sample is not representative of people with clinically disordered BE. Future studies investigating our pathway in people with BE disorder would improve the generalizability of the results.

Moreover, regarding difficulties in emotion regulation, previous studies show that emotion regulation strategies might differ within adulthood. For example, older adults might use more stable or adaptive emotion regulation strategies than younger adults (Riediger and Bellingtier 2022; Eldesouky and English 2018). Although age was entered as the covariate in the study and no significant change was observed, future research should consider a more specific age group when examining difficulties in emotion regulation.

### 4.2 Clinical Implication

Our findings highlight the need for investigating the impact of verbal WRA on the subsequent emotional, cognitive, and behavioral pathologies. Furthermore, because early experiences of verbal WRA may increase the risk for eating problems, based on our findings, helping patients with a history of verbal WRA with efficient self‐regulatory strategies (e.g., developing adaptive emotion regulation strategies and also modifying bias to threatening emotions) may be useful to decrease the risk of the possible appearance of later eating pathologies. Given the limitations of our study, these implications for clinical purposes should be considered with caution.

## 5 Conclusion

This study claims that difficulties in emotion regulation can partly explain the relationship between verbal WRA and BE. Therefore, our findings underline the negative possible outcomes of early verbal WRA in terms of cognitions, emotions, and behaviors, particularly in the field of eating problems.

## Author Contributions

**Elnaz Salemi**: conceptualization, data curation, formal analysis, investigation, methodology, project administration, resources, validation, visualization, writing–original draft, writing–review and editing. **Shaghayegh Zahraei**: conceptualization, supervision, writing–original draft, validation, visualization. **Gholamreza Dehshiri**: supervision, methodology, validation, formal analysis. **Abdolreza Norouzy**: data curation, resources.

## Ethics Statement

All procedures performed in studies involving human participants were in accordance with the ethical guidelines of the institutional research committee and with the 1964 Helsinki Declaration and its later amendments or comparable ethical standards. The ethical standards were discussed and approved by the Research Committee at Tarbiat Modares University (IR.MODARES.REC.1397.086).

## Consent

Informed consent was obtained from all individuals who participated in the study to publish the data.

## Conflicts of Interest

The authors declare no conflicts of interest.

### Peer Review

The peer review history for this article is available at https://publons.com/publon/10.1002/brb3.70054.

## Data Availability

The datasets analyzed during the current study are available in the Mendeley repository, https://doi.org/10.17632/tw753jp85n.1.
